# Supplementary material for: The dynamics of subunit rotation in a eukaryotic ribosome
Source: Biophysica. Author manuscript; Available in PMC 2023 Jul 21. (PMC10361705; doi:10.3390/biophysica1020016)
Supplement: SI results and methods [file NIHMS1898402-supplement-SI_results_and_methods.pdf]

Supplementary Material: The Dynamics of Subunit Rotation in a Eukaryotic Ribosome

Frederico Campos Freitas[1], Gabriele Fuchs[2], Ronaldo Junio de Oliveira[1], and Paul C. Whitford[3,4]

[1]Laboratório de Biofísica Teórica, Departamento de Física, Instituto de Ciências Exatas, Naturais e Educação, Universidade Federal do Triângulo Mineiro, Uberaba, MG, 38064-200, Brazil; fredcfreitas@gmail.com; ronaldo.oliveira@uftm.edu.br

[2]Department of Biological Sciences, The RNA Institute, University at Albany 1400 Washington Ave, Albany, NY, 12222; gfuchs@albany.edu

[3]Department of Physics, Northeastern University, 360 Huntington Ave, Boston, MA 02115; p.whitford@northeastern.edu

[4]Center for Theoretical Biological Physics, Northeastern University, 360 Huntington Ave, Boston, MA 0211

March 31, 2021

1 Supporting Methods

1.1 Force field construction

One of the main features of SMOG models is the definition of stabilizing interactions based on a given structure [18], which defines the structure as the global potential energy minimum. When

working with one structure, force field generation is straightforward and the output files from the SMOG software can be directly used for simulations. Here, the strategy was to create a multi-basin SMOG force field by combining the information of two structures into a single potential, allowing the system to sample transitions between the states. The components of the Hamiltonian responsible for the connectivity and covalent geometry (bonds distances, angles, and dihedrals) were built to have no bias for either classical or rotated structures of the ribosome. The bond distances and angles were assigned the values from the AMBER03 force field [30]. The minimum for each proper dihedral angle was initially obtained from the structures. Although the individual differences may be negligible, the large number of dihedral angles (around 736,600) could lead to the dihedrals introducing a significant bias to one of the endpoints. To avoid this, each dihedral angle used in the force field was defined as the average between the angles found in the two structures.

The contact pairs list for each structure was obtained using the Shadow Map algorithm with default values [31]. The two sets of contacts were merged and their distances were analyzed to check whether they change upon SSU body rotation. The intra-subunit contact distances in the rotated and unrotated structures were compared and a small set change by more than a factor of two. To avoid energetic bias toward either structure, an isoenergetic minimum distance was defined for each intra-subunit contacts. Considering the asymmetry of the Lennard-Jones potential term used to account for the contacts in the force field, the new minimum was defined by:

$$r_n = \sqrt[6]{\frac{2 \cdot \left(r_{\text{rot}}^{-6} - r_{\text{unrot}}^{-6}\right)}{\left(r_{\text{rot}}^{-12} - r_{\text{unrot}}^{-12}\right)}}, \tag{S1}$$

where r_{rot} and r_{unrot} are the pair distances found in the rotated and unrotated structure, respectively. As one may see in the Fig. S1, the potential defined by the new minimum (r_n) would have the same energy ϵ_{iso} at both endpoint distances. The minimum depth is given by ϵ_C

Inter-subunit contact distances that exhibited only small differences upon SSU body rotation were also treated as common contacts. To be treated as common, the inter-subunit contact should

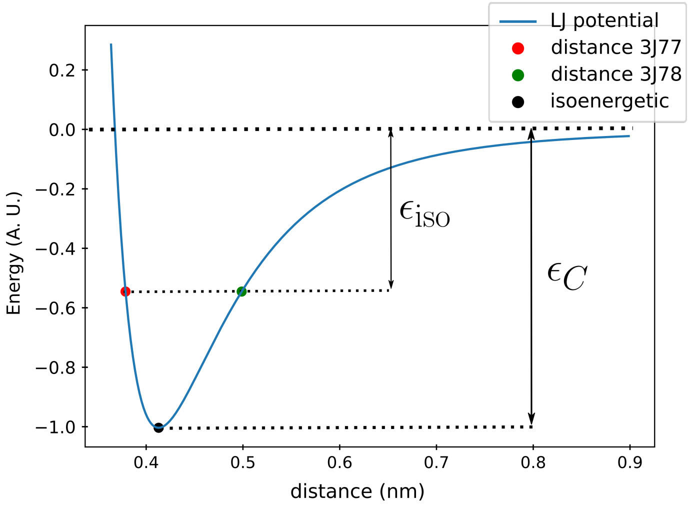

Figure S1: The blue curve shows the Lennard-Jones potential defined by the new distance r_n for a chosen contact pair using the force field term. The red dot is the distance found in the rotated structure (r_{rot}) and the blue dot is the distance in the unrotated structure (r_{unrot}). The new isoenergetic distance (r_n) is defined such that the correspondent distances in both structures have the same energy level. With this definition the new distance has no energetic bias, or is isoenergetic.

have $\epsilon_{\text{iso}}/\epsilon_C > 1/2$ (check Fig. S1). All the common contacts had their weights changed to fit the energy scaling criteria used in SMOG [28]. If the inter-subunit contact distance in the structures differed by more than two-fold, then it was considered to be unique to the rotated/unrotated conformation. For the unique contacts, the minimum (σ_{ij}) was assigned the value found in the conformation from which the contact was found. Preliminary simulations were performed to identify energetic weights for which the rotated and unrotated conformations represent pronounced free-energy minima of comparable depths. The value of the energetic weight used for contacts unique to the rotated configuration was 0.21 and for contacts unique to the unrotated configuration was 0.19.

1.2 Structural Modeling

The structures used in this study represent the classical (unrotated) and hybrid (rotated) states of the yeast ribosome. They are composed of 77 protein chains and 4 ribosomal RNA chains, three in the large subunit (25S, 5.8S, and 5S) and one in the small subunit (18S). They also present an mRNA fragment and are complexed with 1 or 2 tRNAs. The classical structure (PDB 3J78) represents a posttranslocation-like, or pretranslocation-like state of the ribosome, in terms of rotation. However, there are two tRNA molecules present that adopt posttranslocation configurations. The rotated structure (PDB 3J77) presents one tRNA molecule in the hybrid P/E state [18]. To focus on the mechanical properties of the subunits, the tRNA molecules were removed from both models. The aim was to construct a multi-basin SMOG force field that could be used to generate spontaneous and reversible SSU body rotation events. Four structural segments were present in only one of the structures. For segments far from the SSU-LSU interface, the extra atoms were removed. Two segments, one in each structure, are near the SSU-LSU interface and form inter-subunit contacts. In the rotated structure, a section of 25SrRNA (residues 2061 to 2075) was not resolved. On the other hand, the residues 65 to 136 of L24 protein were lacking in the unrotated structure. These two segments were prepared as described in [32,64]. The missing fragments were introduced after applying a least-squares fit of the upstream and downstream residues. The aligned structure is then used to initialize an energy minimization calculation, where all atoms (except for the modeled atoms) were subject to position restraints. The strength of the restraints was decreased after each round of minimization, until the last round was performed with no restraints. The final structures had 206389 non-hydrogen atoms. There was also a region (residues 2526 to 2542 of 25S rRNA and 213 to 225 of protein S1) that was slightly remodeled to improve stereochemistry. The revised model was provided by the Korostelev group.

2 Supporting Results

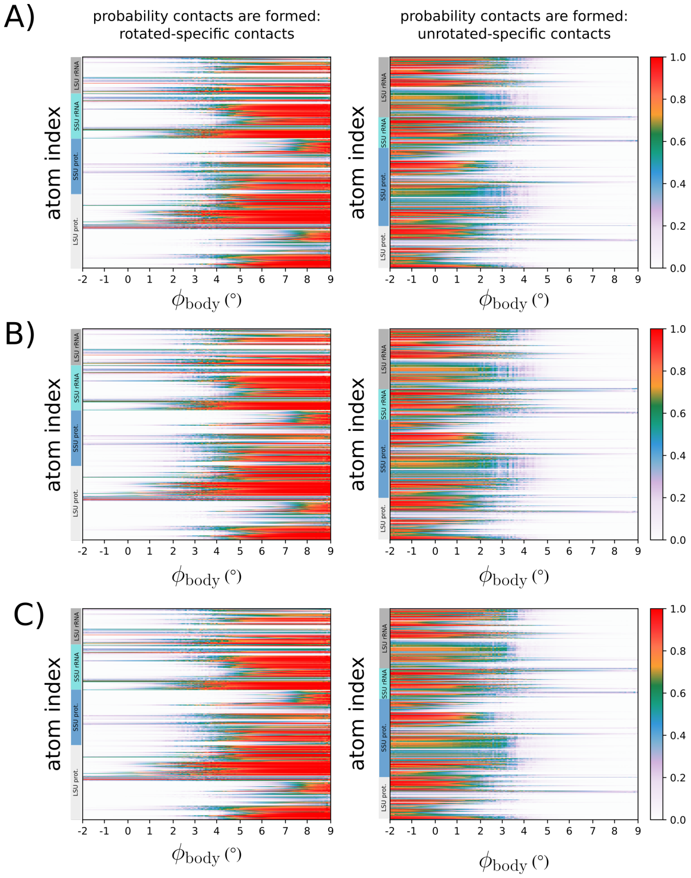

Figure S2: Probability of contact formation for each atom as a function of SSU body angle (ϕ_{body}). The left panels show the probability regarding the unique rotated contacts and the right panels present the probability of unique unrotated contacts being formed. A) Probabilities calculated from the simulation initiated from the rotated configuration. B/C) Probabilities obtained for the two simulations initiated from the unrotated structure. The high degree of similarity between the runs given a set of unique contacts shows the robustness of the model and reproducibility of the results.
